# Publication Charges Associated with Quality Open Access (OA) Publishing and Its Impact on Low Middle Income Countries (LMICs), Time to Reframe Research Policies

**DOI:** 10.31557/APJCP.2021.22.9.2743

**Published:** 2021-09

**Authors:** Mayank Singh, Chandra Prakash Prasad, Abhishek Shankar

**Affiliations:** 1 *Department of Medical Oncology (Lab), Room no 411 4* ^*th*^ * Floor BRAIRCH All India Institute of Medical Sciences (AIIMS) New Delhi, Delhi, India. *; 2 *Department of Radiotherapy, Lady Harding medical college and hospital New Delhi, Delhi India. *

**Keywords:** Open Access (OA), Low middle income Countries (LMICs), publication charges

## Abstract

Dissemination of the scientific literature is as paramount as scientific studies. Scientific publishing has come a long way from localized distribution of few physical copies of journal to widespread and rapid distribution via internet in the 21^st^ century. The evolution of open excess (OA) publishing which has rapidly evolved in last two decades has its heart at the right place with the ultimate goal being timely, and rapid distribution of published scientific work to a wider scientific community around the world and thus ultimately promoting scientific knowledge in global sense. However, quality OA publishing of cancer research involve an average publishing fee of around 1,500 USD which poses a challenge for Low middle income countries (LMICs), where per capita income is low. This has led to deterioration of science in LMICs in the form of publication in Cheap OA predatory journals for sake of securing academic promotions as well as authors ending up paying exorbitant publishing charges out of pocket to get their quality scientific work published. In countries like India and other LMICs, the funding agencies and institution have so far not addressed this problem. Here we assess the framework of open access publishing in LMICs like India and what are the steps which can be taken to facilitate open access publishing in LMICs.

## Introduction

The emergence of open access (OA) publishing stems from a phenomenon that has created a crisis situation faced by the academic institutions around the world called “Serial crisis” in the 1990s. Serial crisis was the result of high subscription cost which eventually led to many libraries being unable to afford these elevated subscription cost and the libraries were left with very few options. Serial crisis had its parallel with the emergence of the world wide web which has revolutionized information sharing across the world, leading to emergence of open access platform for scientific literature. The Association of Research Libraries established the Scholarly Publishing and Academic Resources Coalition (SPARC), in 1997 which was an alliance of academic and research libraries as well as related organizations, to address the serial crisis and promote viable alternatives such as open access.

The first scientific open access platform was founded by Paul ginsparg who developed arXiv registry for archiving physics preprints at Los Alamos National Laboratory which promoted the preprint culture that was existent in high energy physics at that time (Till, 2001). arXiv was a success and now includes papers from varied fields including computer science, synthetic biology etc. Subsequently in 1997 U.S. National Library of Medicine (NLM) made Medline, the most comprehensive index to medical literature on the planet, freely available in the form of Pubmed. Medline was an important step ahead, as it opened up a whole new form of use of scientific literature accessible to both public and professionals alike (Lindberg and Humphreys, 2008). The Journal of Medical Internet Research (JMIR) one of the first open access journals in medicine, was created in 1998, publishing its first issue in 1999 and continues to publish till today demonstrating the importance and acceptance of open access platforms. In 1999, then director of NIH Prof. Harold Varmus proposed a journal called E-biomed with the aim of combining a preprint server with articles that have been peer reviewed which is now known as Pubmed central which comprises of peer reviewed post print articles. In the year 2000, NIH floated Pub Med central, An open access platform for NIH funded research which has continued to grow exponentially and is a major source of dissemination of NIH funded research around the globe. 

*The framework of open access publishing*

The root of OA publishing lies in the serial crisis which led to the emergence of OA platform in the 2000’s. The scientific community felt the need to have a framework to guide the implementation of open access publishing. The first initiative in this direction was the Budapest open access initiative (BOAI) at a conference convened by open society institute in late 2001, which was represented by group of academic researchers and its affiliates to promote open access policies by deposition of referred journals to open access platforms and development of OA journals accessible to all without the pay per view policy. BOAI is generally regarded as a defining event in history of OA. BOAI was followed by Bethesda statement of OA initiative which was issued at a meeting held at Howard Hughes Medical Institute. It further defined the elements of OA publishing by defining how users will share OA and license granting rights for the reuse of the work. (Crawford, 1992). In a follow up the Budapest Open Access Initiative in 2002, and the Bethesda Statement on OA Publishing in 2003, the Berlin Declaration is regarded as the third event which defined the OA movement which was drafted at conference held by the Max Planck Society in October 2003 which was a huge gathering of many cultural and political societies. Overall these three events defined the principal and values of open access publishing with a common theme that scholarly publishing should be free and accessible to all and the cost associated with this publishing should be taken care by the scientists and associated funding agencies/Institutes, Such a model will relieve the pressure on the institutions and will address the problem that arose due to serial crisis. The journals can operate as exclusively open access journals or can function in a hybrid mode by offering the author OA choice as well as traditional publishing. 

In general all of these declarations defined two types of open access: Firsts is the Green OA in which the manuscripts can be put in institute repository or authors website which will be different then the final edited published version of the manuscript and this final version is subjected to period of embargo as determined by the publishing house following which the work can be placed on online platforms like research gate etc. Second and the most popular OA is Gold OA in which the authors, Funding agencies or institution cover the publication charge. Gold OA has gradually evolved as the most popular form of OA. In early 2000, Biomed central which is a for profit publishing house was launched by current science group which now has many prominent open access cancer research journals like Molecular Cancer and BMC Cancer etc. PloS was another commercial publishing house that emerged during these times with PLoS Biology and subsequently PLoS Medicine and PLoS one. 

*Plan S (Plan Science) and its impact on scientific publishing*

Plan S is the recent phenomenon in scientific publishing and has been framed by the coalition of funding agencies in Europe. cOAlition S an international consortium of research funding and performing organisations has take action towards implementation of Plan S. It underlines the fundamental principles of open access publishing in modern times as OA publishing has come a long way since Budapest, Berlin and Bethesda declaration which were framed in early 2000s. It states that no science should be locked behind paywalls. Plan S does not stress upon any particular OA business model and therefore publishers have a set of principals on which they can rely upon. 

Plan S states the fundamental principles for future OA publishing. As per Plan S Science Europe, funders, the European Research Council and the European Commission will work together to clarify and publish implementation details. Plan S states that “With effect from 2021, all scholarly publications on the results from research funded by public or private grants provided by national, regional and international research councils and funding bodies, must be published in OA Journals, on OA Platforms, or made immediately available through OA Repositories without embargo” (Plan S. https://www.coalition-s.org. Updated 2019. Accessed May 4, 2021). The aims of plan S is to have Scientific knowledge which is free from all boundaries but the global applicability of Plan S is still a question that needs to be addressed specifically for scientific community working in developing economies. Plan S has its limitation if it is applied in perspective of LMICs specially in view of pre existing funding crunch with research being at the lower end of financial spectrum of the government. The obvious question arises who is going to pay for the publication of research in quality OA journal as many governments in LMICs don’t consider research as a priority so Research publications are still a far cry for them. Recently the trend in LMICs has shifted to promoting research that can have consequences in near future which has discouraged the basic research which is the corner stone of innovation in long term. This trend needs to change and policymakers need to understand that promoting quality basic research with its subsequent dissemination using Quality OA platform is important for any country in the long term. The importance of such approach can be seen in the outbreak of SARS Cov2 epidemic where countries like USA and many European countries who have already promoted RNA vaccine development in 90s with no immediate benefits have been able to contain the virus more effectively as compared to countries like India where people have tremendously suffered due to lack of scientific policies and infrastructure. The rapid development of safe and effective vaccine was possible due to preexisting knowledge of SARS viruses and as well as mRNA Vaccine technology (Verbake et al, 2021).

*High cost of Open access/ No open access publishing in cancer research and its impact on publishing quality*

As of today many prominent traditional publishing house like Cell Press have launched exclusive open access cancer journals like Cell reports, iScience, Heliyon as well as new publishing houses with exclusive open access journals like MDPI, Frontiers have emerged. However it should be remembered that many prominent journal in both basic and clinical cancer research which operate in hybrid mode like Cancer cell, Journal of clinical Oncology, JAMA Oncology, Journal of National Cancer institute, Molecular cancer, Clinical cancer research, Cancer research etc have very high cost of publishing as listed in Table 1 which ranges anywhere between 1,000 to 9,500 USD per article. In Table no 1, we have listed top ten oncology journals as per google scholar (https://scholar.google.co.in/citations?view_op=top_venues&hl=en&vq=med_oncology) with search term oncology and listed the cost associated with open access publishing in these journals. It is clear that for or a research group who is working in a developing economy like India or other low-income country of south Asia, OA publishing is a dual problem. Firstly the exchange rates are very high for western currency in these countries and secondly funding agencies as well as institutes just don’t have provisions to cover for these charges. The government aided funding agencies in India like DBT (Department of Biotechnology), DST (Department of Science and technology), ICMR (Indian Council of Medical Research) etc have just not kept themself in pace with the rapid changing world of publishing scientific literature and as a result there is no provision of covering these publishing charges in the existing framework of grant system as well as institutional academic setup.

*The culture of publish or perish world and its relevance in developing countries*

The conception of ‘Publish or Perish’ has been a central doctrine in academia, especially in high-income Western countries for years. Its effects are apparent as the rate of academic publishing continues to grow exponentially. Faculties, Researchers and Scientists at the academic institution believe that strong research and publication track are necessary in their academic review and promotion (Green and Baskind, 2007; Youn and Price, 2009; Harley et al., 2010). Moreover, faculties and scientists are apprehended by the quantity and publication type (peer-reviewed, prestigious journals, journals with high impact factor etc) expected from them. In developing countries like India, PhD or Master degree students look towards good academic Institutes in USA and Europe for their Post Doctoral training or PhD degree. Because of good infrastructure and funding in West, they publish decent amount of publication in peer-reviewed and high impact journals, within a span of 4-5 years. The main reason behind their publication success stems from a good amount of Research/publication money which is provided in the granted projects/or by institutional support. In India, for a faculty position a candidate is suppose to have international postdoctoral exposure with good publication records, so getting a job for individual with good publication record is not a problem. However, after joining , such faculties and scientists are not able to maintain their good publication track, mainly due to lack of publication support by the grant agencies, even after carrying out good research work. The great benefit of publishing in OA journal is association with increased citation index that plays a key role in uplifting individual research work as well as fostering international collaboration (Adler et al., 2009). In our experience, we have observed that cancer researchers working as faculties and scientists in developing countries like India, find it difficult in publishing in OA journals (due to high publication charges), hence funding agencies should device strategies to support publication costs in sanctioned grants. 

*Paid OA/ Quality publishing and predatory journals where to draw a line*

“Predatory publishers” is the term coined by Jeffery Beall about a decade back, for the publishers who circulate fake journals and take advantage of the open-access model with deceitful practices (Beall, 2012). Such journals has been given various names like fake journals, predatory journals, and many others. These journals influence and exploit the open access publishing model but leave out the quality checks and editorial scrutinise that are normally provided by genuine or legitimate journals, such as peer review process, plagiarism recognition, and authentication of ethical approval of experiments. The major goal of these fake journals is to generate revenue from article processing charges, and therefore have modest consideration for the scientific quality or integrity of the work they accept. They also promise expedient rapid publication, review process, and relatively low author processing charges/or publication fees, compared to legitimate OA journals (Moher and Moher, 2016). In all, Predatory journals are damaging the quality of open access (OA) Journals. Hence, traditional journals are convincing researchers to avoid OA routes so they might not fall in predatory journals trap. In LMICs, where researchers or scientists are not exposed to international research due to inland education often get entrapped in predatory journals web, and when the true nature of these journals come to light, it interferes with their academic jobs or promotion. In oncology, a recent study identified 281 potential predatory journals and 222 legitimate journals from bibliographic databases like PubMed/MEDLINE, and Science Citation Index/Science Citation Index Expanded (SCI/SCIE) (Abdelhafez et al., 2019). The damaging effect of Predatory OA publishing was demonstrated by a 2013 paper by John Bohannon in which they submitted fake scientific papers to 304 journals which were so called paid OA publishers, The articles were designed in such a way that the scientific flaws in the submitted manuscripts should have led to their immediate rejection at editor or reviewer level but as per the data provided in this landmark paper about 60% of journal accepted them which lay bare the non-existent or substandard peer review policies in these journals. What was more disturbing was that among the journals, who accepted the fake manuscript near about 80% were operating from India (Bohannon, 2013).

*Current scientific policies in India and what needs to be changed*

Till date, there are no strong policies devised by the government funding agencies or by the educational institutes that exists in supporting the OA framework in LMICs including India. In the average extramural funding grants (in India), a contingency money is provided (approx-0.5.to 1% of the total grant) that will be around 1 lakh/year (~1300 USD). Furthermore, contingencies apart from publication fees includes other expenditures like registration fees for conference, membership for scientific societies, stationary spending etc. So, it is difficult to pay for average OA charges with such a nominal amount. As most extramural grants are for three years, by the time research article from that project come into pictures theses funding are long gone (including final submission of budgets and utilizations to funding agencies). Funding agencies however don’t have any provision for separate allocation for publication money, after the project has been successfully submitted. Due to all these hurdles, majority of Indian authors cover their expenditure for OA out of their pockets, and that to in predatory journals (Seethapathy et al., 2016), as they are affordable compared to the legitimate publishing houses like Frontiers, MDPI, Impact Journals etc. 

We have some recommendations that might be helpful for government aided funding agencies and educational institutes in supporting OA charges:

1. Publication money (cost for at least one OA article) should be allocated to every extramural grant provided to the Principle Investigator (PI), with a clause that money should be used within 5 years from the date of start of the project. This money should either be given to the PI directly or he can claim from funding agencies as and when required (during these 5 years).

2. Government should promote funding agencies to tag itself with legitimate publication houses that provide OA, where research articles can be submitted by PI without paying or discounted OA charges. Similar platform has been developed between NIH and Oxford journals.

3. Government aided/or public institutes should also formulate policies to publish OA articles coming from their institutional/or intramural grants. For example, each year amount has to be fixed by administration for the allocation of publication charges for OA journals. Departments that are actively involved in research can claim such funds for publishing. Just like research funds this money should not come under income-tax bracket, hence promoting research.

4. Funding agencies should also plan to advertise special calls, where PI can claim OA charges that has already been paid from their pockets for the research articles published in legitimate OA platforms, from the specific grant provided to PI.

Recently a few steps have been taken by Indian Government to revamp India Science policy called Science Technology and Innovation policy 2020. An Expert group has came up with some recommendations with predominantly one recommendation having a bearing on paying APCs according to which authors can pay APCs through the sanctioned grant for publication in journal of repute. It further proposes a system through which government can be directly invoiced and a central payment system for APCs is being done (Mallapaty, 2020), Though this policy is still not implemented but if it is implemented in timely manner it will have far reaching positive consequences on research in India.

In conclusion, there are no doubts that OA enhances visibility and impact of research work as OA articles are downloaded and cited more often, than articles from non-OA or traditional journals. Free access to scientific information is key to knowledge. Researchers in LMICs and small research institutions should have access to all Open Access articles but a simultaneous push to promote quality OA should be practised. Open Access articles are published faster and reach broader audiences, compared to non-Open Access journals. Hence, for the developing countries like India, policies should be formulated to cover the OA charges as no such provision are available in the current policy framework. In the present article, we have touched upon the policies that need to be implemented regarding publication charges coverage in developing countries and how they can be implemented. Such policies will not only boost the research activities but also make researchers to not fell into the trap of predatory journals. 

**Table 1:** Top Ten Oncology Journals as Per Google Scholar https://scholar.google.co.in/citations?view_op=top_venues&hl=en&vq=med_oncology) and Open Access Publication Fees Associated with Them. Source (Access date 4th May 2021)

S No	Name of Journal	Publisher	Publication fees discount and waiver to Low income economies	Publication Charges as of April 2021	Impact factor as of April 2021
1	Journal of Clinical Oncology	American society for Clinical Oncology (ASCO)	None	5500 USD (OA fee) ^a^	32.95
2	Lancet Oncology	Elsevier	None	5000 USD (OA fee) ^b^	33.75
3	Clinical cancer Research	American Association for Cancer research (AACR)	Yes (25% to 100%)	3300 USD (OA fee) ^c^	10.1
4	Nature Reviews Cancer	Springer Nature	None	9500 USD (OA fee) ^d^	53.03
5	Annals of Oncology	Elsevier	None	3200 Euro (OA Fee) ^e^	18.27
6	Cancer Cell	Cell Press	None	8900 (OA fee) ^f^	15.36
7	Cancer Research	American Association for Cancer research (AACR)	Yes (25% to 100%)	3300 USD (OA fee) ^c^	9.72
8	Oncotarget	Impact group	None	3400 (OA fee) ^g^	Not available
9	JAMA Oncology	American medical association (AMA)	None	3000 (OA fee) ^h^	24.79
10	Cancer Discovery	American Association for Cancer research (AACR)	Yes (25% to 100%)	6000 (OA Fee) ^c^	29.49

^a^, https://ascopubs.org/jco/authors/open-access; ^b^, https://www.thelancet.com/lancet/about; ^c^, Publication Fees and Reprints | American Association for Cancer Research (aacrjournals.org); ^d^, https://www.nature.com/articles/d41586-020-03324; ^e^, https://www.elsevier.com/journals/annals-of-oncology/0923-7534/open-access-options; ^f^, https://www.cell.com/rights-sharing-embargoes; ^g^, https://www.oncotarget.com/editorial-policies/#publication-fees; ^h^, https://jamanetwork.com/journals/jamaoncology/pages/for-authors.

## Author Contribution Statement

Study conception and design: MS, CPP; Draft manuscript preparation: MS, CPP; Final Editing: MS, CPP and AS. All authors reviewed and approved the final version of the manuscript.

## References

[B1] Abdelhafez I, Alahmad YM, Skenderi F (2019). Predatory Journals in Oncology: A Critical Appraisal. Conference proceeding ‘2nd World Congress on Undergraduate Research’ at Oldenburg.

[B2] Adler R, Ewing J, Taylor P (2009). Citation Statistics A Report from the International Mathematical Union (IMU) in Cooperation with the International Council of Industrial and Applied Mathematics (ICIAM) and the Institute of Mathematical Statistics (IMS). Statist Sci.

[B3] Beall J (2012). Predatory publishers are corrupting open access. Nature.

[B4] Bohannon J (2013). Who’s Afraid of Peer Review?. Science.

[B5] Crawford W, ISBN 9780838911068, pp 11–5 (1992). Open access : what you need to know now.

[B6] Green RG, Baskind FR (2007). The second decade of the faculty publication project: Journal article publications and the importance of faculty scholarship’, Journal of Social Work Education. Routledge.

[B8] Lindberg DAB, Humphreys BL (2008). Rising Expectations: Access to Biomedical Information”. Yearb Med Inform.

[B9] Mallapaty S (2020). India pushes bold ‘one nation, one subscription’ journal-access plan. Nature.

[B10] Moher D, Moher E (2016). Stop predatory publishers Now: Act collaboratively. Ann Intern Med.

[B11] Seethapathy GS, Santhosh Kumar JU, Hareesha AS (2016). India’s scientific publication in predatory journals: need for regulating quality of Indian science and education. Curr Sci.

[B12] Till JE (2001). Predecessors of preprint servers. Learned Publishing..

[B13] Verbeke R, Lentacker I, De Smedt SC (2021). The dawn of mRNA vaccines: The COVID-19 case. J Control Release.

[B14] Youn T, Price T (2009). Learning from the experience of others: The evolution of faculty tenure and promotion rules in comprehensive institutions. J Higher Edu.

